# Rescue ALPPS: Intraoperative Conversion to ALPPS during Synchronous Resection of Rectal Cancer and Liver Metastasis

**DOI:** 10.1155/2014/487852

**Published:** 2014-11-24

**Authors:** Terence Jackson, Kelly A. Siegel, Christopher T. Siegel

**Affiliations:** ^1^Department of Surgery, University Hospitals Case Medical Center, 11100 Euclid Avenue, Cleveland, OH 44106, USA; ^2^Division of Hepatobiliary and Transplant Surgery, Department of Surgery, University Hospitals Case Medical Center, Cleveland, OH 44106, USA; ^3^Liver Center for Excellence, UH Digestive Health Institute, University Hospitals Case Medical Center, OH 44106, USA; ^4^Case Western Reserve University School of Medicine, OH 44106, USA

## Abstract

Future liver remnant (FLR) is the most important deciding factor in planning for liver resection. Portal vein embolization (PVE) was first introduced in the 1980s to induce liver hypertrophy, enabling removal of multiple/bilobar tumors. PVE was later combined with sequential hepatectomies with the aim of allowing the liver remnant to hypertrophy (15–20%) between procedures. However, the interval between the two procedures (3–8 weeks) put patients at risk for disease progression. With portal vein ligation alone or when combined with sequential hepatectomy, there is also a risk for inadequate liver hypertrophy because of intrahepatic portal collaterals leading to a high (19–30%) dropout rate. The ALPPS procedure (associating liver partition and portal vein ligation for staged hepatectomy) was recently developed as a feasible means to perform extensive/bilobar liver resections. It produces rapid, enormous hypertrophy of the remnant, making previously unresectable lesions resectable. Indications for ALPPS include any extensive liver resection with inadequate FLR. Here we present a novel indication for ALPPS as a rescue when inadequate FLR was faced intraoperatively, during a simultaneous resection of rectal primary and liver metastasis.

## 1. Case Report

**Figure 1:**
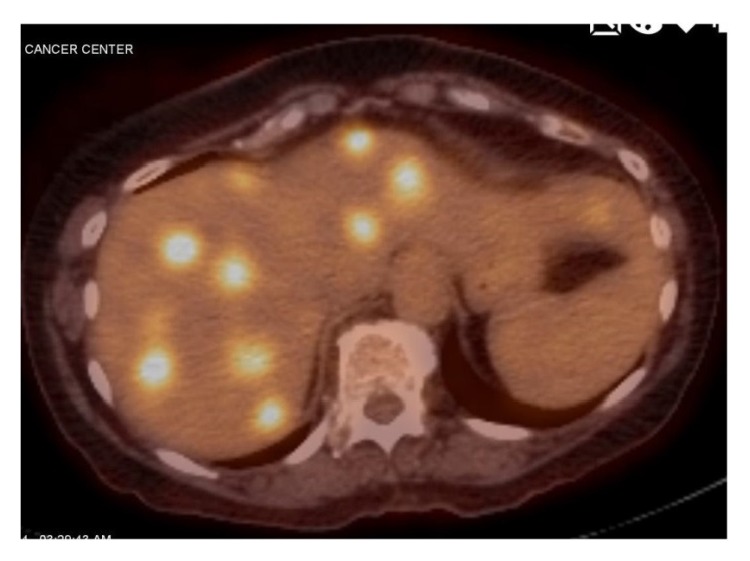
Preoperative PET.

An 80-year-old female had presented with back pain, 6-7 months of intermittent bloody stool, and diarrhea. Preliminary imaging showed multiple ill-defined lesions involving all segments of the liver, mural thickening of the rectum with eccentric narrowing of the lumen, and multiple enlarged perirectal lymph nodes. Colonoscopy showed a partially obstructing, infiltrative rectal mass about 10 cm from the anus. She was presented at a multidisciplinary tumor board. The board's recommendations included staging workup (including MRI pelvis, MRI liver, and PET scan [representative cut see in Figure 1]), neoadjuvant chemotherapy, and evaluation by hepatobiliary and colorectal surgery. Liver MRI showed a dominant metastatic lesion 3.5 × 3.5 cm in segment 8, multiple other lesions including one in segment 2, one in segment 3, two in segment 4A, 2 in segment 4B, and multiple lesions in segments 5, 6, and 7. She did not have any derangements in liver function and had an albumin of 4.0.

**Figure 2:**
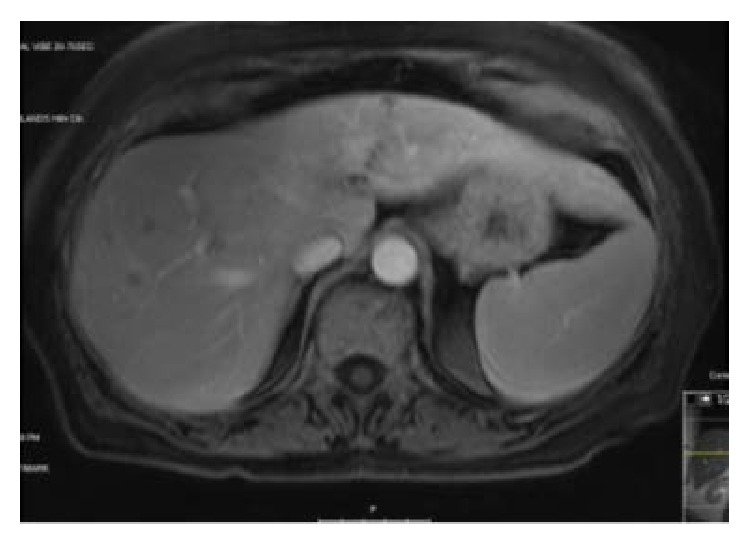
Postchemotherapy MRI.

She underwent 4 cycles of FOLFOX chemotherapy and was reimaged to evaluate for response. Imaging showed good response to chemotherapy in both liver and rectal lesions (as seen in Figure 2). She underwent 2 more cycles of FOLFOX.

She was reevaluated by our hepatobiliary and colorectal surgeons, who felt that her disease may be amenable to resection of the primary lesion and a combination of resection and ablation of liver metastasis. Patient underwent preoperative pelvic radiation (25 Gy in 5 doses) prior to a planned combined procedure.

*Operative Course*. Day 0: Anterior proctosigmoidectomy, coloanal anastomosis, diverting ileostomy, partitioning of the right lobe of the liver with right portal vein ligation (ALPPS Step 1).

An anterior proctosigmoidectomy with coloanal anastomosis was performed first. Intraoperative ultrasound was used to evaluate the liver lesions, found scattered throughout the right and left lobes. Using ultrasound localization, two lesions in segment 5, two lesions in segments 4A and 4B each, one in segment 3, and one in segment 2 were ablated using a 2 cm microwave ablation needle for 10 minutes at 45 watts. At this time, the right liver was reevaluated. Originally, the plan was to resect the right lobe. However, given the extent of ablation on the left side, if we had proceeded with the trisegmentectomy, an inadequate FLR would have been unavoidable. A conventional 2-stage hepatectomy was a possible option. However, given the heavy tumor burden in the right liver and the extent of ablation on the left, it was unclear how much oncologic benefit a physiologically safe 2-stage hepatectomy would have. At this point, we decided to proceed with an ALPPS procedure. A cholecystectomy was first performed. Right lobe of the liver was mobilized and short vessels draining the liver into the vena cava were ligated such that the only vessel draining the right lobe was the right hepatic vein. Portal structures were then dissected to isolate the right hepatic artery, right portal vein, and right bile duct. The right hepatic artery and right bile duct were left intact; the right portal vein was divided using an endovascular GIA stapler with a 2.5 mm load. A Pringle maneuver was then performed for 10 minutes at a time and the liver parenchyma was divided using an Erbe water dissector and the Caiman energy device. This was continued between large traversing middle hepatic veins and its branches, which were divided using the stapler. Once the parenchyma was completely transected, the surfaces were treated with argon beam coagulation and then fibrin glue. The liver was then wrapped in plastic to prevent adhesion and the abdomen was closed. Following this procedure, the patient was extubated, with no hemodynamic instability and moved to intensive care.

*Postoperative Course*. She required 2 units packed red blood cell transfusion for acute blood loss anemia on POD 5; however, the remainder of the postoperative period was uneventful. CT volumetrics on POD 9 showed a total liver volume of 1690 cc with the left liver estimated to comprise approximately 40–50% of the total volume.

**Figure 3:**
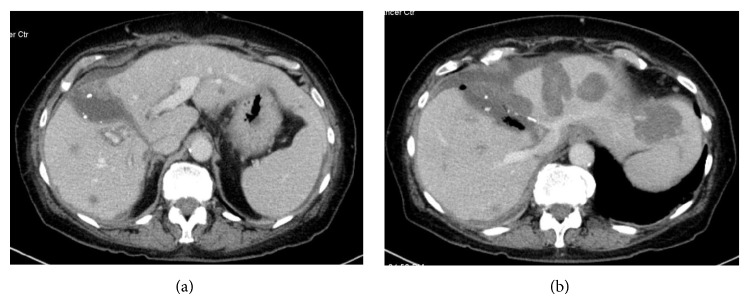
Post-FLR hypertrophy.

For day 9 imaging, see Figure 3.

*Operative Course*. Day 10: Completion right hepatectomy (ALPPS Step 2).

The abdomen was reexplored using the existing incision. Plastic sheets covering the liver were removed and a Doppler examination was performed which showed intact blood flow in the right hepatic artery and vein. A questionable lesion in segment 3 was biopsied and demonstrated a hemangioma with no evidence of cancer. The right hepatic artery was ligated and divided. The right bile duct and right hepatic vein were divided using a GIA stapler. The right lobe was delivered via the abdominal incision and the abdomen was closed. Patient was extubated, hemodynamically stable and returned to regular nursing floor.

**Figure 4:**
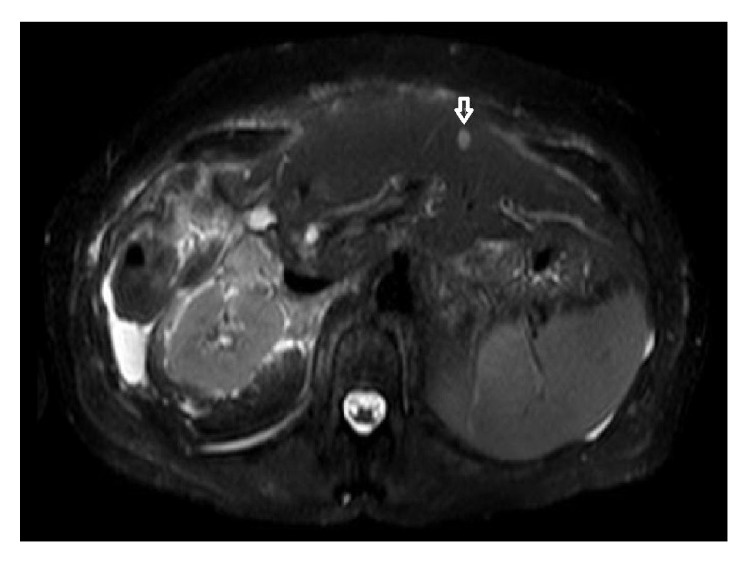
Postoperative follow-up MRI (small white arrow indicates new suspicious lesion).

*Postoperative Course*. Patient had an uneventful recovery and was discharged to home on postoperative days 16 and 10. On her first postoperative follow-up visit, her Total bilirubin was 2.0 and ALT/AST/ALP were 63/61/294. Of concern, her postoperative liver MRI showed interval development of a new, T2 hyperintense, nonenhancing lesion (too small to characterize), along with an increase in CEA from 14.0 to 16.4 (Figure 4, small white arrow).

## 2. Discussion

ALPPS is a recently developed 2-step curative hepatectomy for extensive/bilobar disease, which produces rapid hypertrophy of FLR (up to 74% in ~7 days). Since Lang presented the first series of 3 cases at E-AHPBA in April, 2011, approximately 350 cases have been added to the registry [1, 2]. Portal vein ligation redistributes hepatotrophic factors to the FLR and liver partitioning, not only disrupting cross-portal circulation between the diseased hemiliver (DH) and the remnant but also producing a massive inflammatory response (regenerative stimulus) leading to release of growth factors. This has a profound effect on liver regeneration. This rapid hypertrophy enables surgeons to proceed with the second hepatectomy between 1 and 2 weeks from the first procedure, thus giving patients a chance of curative resection in a single hospital stay for liver disease that would otherwise have been considered unresectable by means of a 2-stage procedure. Up to 200% hypertrophy has been described after longer intervals between procedures. During the 1-2-week interval between first and second steps, the deportalized liver provides auxiliary metabolic support during FLR hypertrophy.

ALPPS has been described as a method of rescue in patients who fail to achieve adequate FLR hypertrophy after portal vein embolization. In our case, ALPPS served as a rescue when, intraoperatively, an inadequate FLR was found to be unavoidable. It allowed us to obtain enough FLR over the course of 9 days to safely perform a trisegmentectomy and allowed the patient to recover without complications.

Main drawback of the ALPPS procedure is a mean morbidity of 43–60% with a mean mortality of approximately 15–20% [3]. Most common complications include postoperative liver failure, deep space infections, and sepsis secondary to bile leaks from the resection bed and necrosis of a devascularized segment IV. Despite these complications the general consensus is that published morbidity and mortality is secondary to a steep learning curve and is likely to improve over time.

One of the biggest concerns with the ALPPS approach is its overall oncologic benefit. Recently published data has shown up to 20% recurrence rates at 6-month follow-up [4]. Aloia and Vauthey raised the concern that portobiliary manipulation during the first surgical step and leaving the tumor behind was against the “no-touch” technique. Increased tumor handling during the first step and subsequent aggressive liver hypertrophy may stimulate tumor metastasis and growth [5]. Authors have reported recurrence of liver tumors in 86% (6 out of 7) and lung metastases in 42% (3 out of 7) of patients, despite R0 resection [6, 7]. Our patient presented at one-month follow-up with a suspicious lesion on segment II (too small to be characterized) and a slight elevation in CEA. She continues to do well clinically, but how her cancer will behave and what this lesion will turn out to be over time remain to be seen. ALPPS is one of the most fascinating advances made in liver surgery, though many questions still remain unanswered. As we grow in experience and number, we hope this significantly improves overall patient outcomes.
